# Germline Mutations Related to Primary Hyperparathyroidism Identified by Next-Generation Sequencing

**DOI:** 10.3389/fendo.2022.853171

**Published:** 2022-04-28

**Authors:** Hye-Sun Park, Yeon Hee Lee, Namki Hong, Dongju Won, Yumie Rhee

**Affiliations:** ^1^ Department of Internal Medicine, Gangnam Severance Hospital, Yonsei University College of Medicine, Seoul, South Korea; ^2^ Department of Internal Medicine, Seoul Eco Internal Medicine Clinic, Seoul, South Korea; ^3^ Department of Internal Medicine, Endocrine Research Institute, Yonsei University College of Medicine, Seoul, South Korea; ^4^ Department of Laboratory Medicine, Severance Hospital, Yonsei University College of Medicine, Seoul, South Korea

**Keywords:** sporadic primary hyperparathyroidism, familial primary hyperparathyroidism, parathyroid cancer, variants of unknown significance (VUS), next-generation sequencing, germline mutation

## Abstract

Primary hyperparathyroidism (PHPT) is characterized by overproduction of parathyroid hormone and subsequent hypercalcemia. Approximately 10% of PHPT cases are hereditary, and several genes, such as *MEN1*, *RET*, *CASR*, and *CDC73*, are responsible for the familial forms of PHPT. However, other genetic mutations involved in the etiology of PHPT are largely unknown. In this study, we identified genetic variants that might be responsible for PHPT, including familial PHPT, benign sporadic PHPT, and sporadic parathyroid cancer, using next-generation sequencing (NGS). A total of 107 patients with PHPT who underwent NGS from 2017 to 2021 at Severance Hospital were enrolled. We reviewed the pathogenic variants, likely pathogenic variants, and variants of uncertain significance (VUS) according to the American College of Medical Genetics and Genomics and the Association for Molecular Pathology criteria. Of the 107 patients (mean age: 47.6 ± 16.1 years, women 73.8%), 12 patients were diagnosed with familial PHPT, 13 with parathyroid cancer, and 82 with benign sporadic PHPT. Using NGS, we identified three pathogenic variants in two genes (*CDC73* and *MEN1*), 10 likely pathogenic variants in six genes (*CASR*, *CDC73*, *LRP5*, *MEN1*, *SDHA*, and *VHL*), and 39 non-synonymous VUS variants that could be related to parathyroid disease. Interestingly, we identified one *GCM2* variant (c.1162A>G [p.Lys388Glu]) and five *APC* variants that were previously reported in familial isolated hyperparathyroidism, benign sporadic PHPT, and parathyroid cancer. We also analyzed the characteristics of subjects with positive genetic test results (pathogenic or likely pathogenic variants), and 76.9% of them had at least one of the following features: 1) age < 40 years, 2) family history of PHPT, 3) multiglandular PHPT, or 4) recurrent PHPT. In this study, we analyzed the NGS data of patients with PHPT and observed variants that could possibly be related to PHPT pathogenesis. NGS screening for selected patients with PHPT might help in the diagnosis and management of the disease.

## Introduction

Primary hyperparathyroidism (PHPT) is a common endocrinological disorder with an estimated prevalence of one to seven per 1,000 adults (1). It is characterized by overproduction of the parathyroid hormone (PTH) and hypercalcemia, leading to complications such as osteoporosis and formation of renal stones. (2) The hereditary form of PHPT accounts for approximately 10% of all cases, including multiple endocrine neoplasia (MEN) 1, MEN2A, familial hypocalciuric hypercalcemia (FHH), neonatal severe hyperparathyroidism, hyperparathyroidism jaw tumor syndrome (HPT-JT), and familial isolated hyperparathyroidism (FIHP). (3, 4) Eighty-five percent of PHPT cases are usually sporadic, and < 1% of PHPT cases present as parathyroid cancer, which is commonly associated with severe hypercalcemia and associated clinical manifestations (4).

Several genes have been established as containing disease-causing mutations for the familial PHPT: *MEN1* gene for MEN1, *RET* gene for MEN2A, *CASR* gene for neonatal severe hyperparathyroidism, and *CDC73* gene for HPT-JT. A few other genetic mutations in familial PHPT have been discovered relatively recently. FHH was initially known to be caused by a mutation in *CASR*. However, *GNA11* and *AP2S1* mutations were additionally identified as causes of FHH type 2 and type 3, respectively. (5, 6) In addition, *GCM2* mutations were recently identified in FIHP, another form of familial PHPT. Several germline mutations in *CDC73*, *MEN1*, *CASR*, and *PTH* are associated with benign sporadic PHPT or parathyroid cancer. (7) However, data on genetic abnormalities in PHPT are limited. In this study, we identified genetic alterations that may be involved in the pathogenesis of PHPT, using next-generation sequencing (NGS) data.

## Methods

### Study Participants

We enrolled 107 patients with PHPT who visited the endocrinology clinic at Severance Hospital and underwent NGS from 2017 to 2021. PHPT was diagnosed as inappropriately high intact PTH (normal range: 15–65 pg/mL) with normal or high albumin-corrected serum calcium levels (normal range: 8.5–10.1 mg/dL). We excluded patients who had elevated PTH levels due to secondary causes, such as chronic kidney disease and vitamin D deficiency. We collected baseline information of the study participants, including age, sex, medical history, family history, and PHPT forms. We classified MEN, FHH, and FIHP as familial PHPT. (8) Persistent PHPT was defined as elevated serum calcium levels within 6 months after primary surgery for PHPT, whereas recurrent PHPT was defined as elevated serum calcium levels that presented after 6 months of initial normocalcemia following primary surgery for PHPT. (9) Multiglandular PHPT was defined as the presence of two or more enlarged parathyroid glands. This study was approved by the Institutional Review Board of Severance Hospital, Yonsei University Health System, Seoul, Korea (No.4-2021-1387).

### Laboratory Data and Gene Sequencing

Calcium, phosphorus, albumin, alkaline phosphatase, intact PTH, blood urea nitrogen, creatinine, 25-hydroxy vitamin D, ionized calcium, and 24 h urinary calcium levels were measured routine laboratory methods. Albumin-corrected calcium was calculated by the following equation: serum calcium (mg/dL) + 0.8×(4.0-alubmin [g/dL]). (10) The serum intact PTH concentration was measured using a second-generation PTH assay (Elecsys PTH; Roche Diagnostics, Mannheim, Germany) on a Cobas e801 immunoassay analyzer (Roche Diagnostics).

The patients underwent gene sequencing using either targeted gene sequencing or clinical exome sequencing. A customized NGS panel was used for targeted sequencing, which included 400 genes related to various endocrine disorders. (**Supplemenatry Table 1**) The other NGS panel was the xGen Inherited Diseases Panel (Integrated DNA Technologies, Coralville, IA, USA) comprising 4,503 genes for clinical exome sequencing. We used this expanded NGS panel since September 2019, and the patients who visited the clinic before September 2019 underwent targeted gene sequencing.

Genomic DNA was extracted from leukocytes of peripheral blood samples using the QIAamp Blood DNA Mini Kit (Qiagen, Hilden, Germany) following the manufacturer’s instructions. Subsequent sequencing procedures and data analyses were conducted as previously described. (11, 12) The variants were interpreted using the 5-tier classification system recommended by the American College of Medical Genetics and Genomics and the Association for Molecular Pathology guidelines (13).

### Data Analysis

We analyzed all reported pathogenic variants, likely pathogenic variants, and variants of uncertain significance (VUSs). Pathogenic and likely pathogenic variants were defined as positive genetic tests. Among VUSs, we prioritized variants that met the following: 1) non-synonymous variants that are missense variants, frameshift variants, or variants at canonical ± 1 or 2 splice sites and 2) variants in genes that were in the candidate gene list of parathyroid disease. We built a list of genes associated with parathyroid disease based on previous studies. We obtained a list of candidate parathyroid genes from Cetani et al. (14) They sorted 118 genes co-occurring with the term ‘parathyroid’ in literature-supported statements from the GeneRIF Biological Term Annotations dataset, and 41 genes from previous studies. (15–17) We then collected candidate genes from previous studies on PHPT. (14, 18) **Supplementary Table 2** shows the final 161 candidate gene list of parathyroid disease.

### Statistical Analysis

Values are presented as mean with standard deviation for normally distributed continuous variables, or median with interquartile range for non-normally distributed continuous variables. Categorical variables are described as numbers with percentages (%) and compared using the chi-square analysis. The Mann–Whitney test was used for continuous variables with non-normal distribution to compare the differences between groups. Statistical significance was set at p < 0.05. All statistical analyses were conducted using the Statistical Package for Social Sciences for Windows version 26.0 (IBM Corp., Armonk, NY, USA).

## Results

The baseline characteristics of the study subjects are shown in **Table 1**. The mean age of the study subjects was 47.6 ± 16.1 years and 73.8% of them were women. Of the 107 study patients, 12 (11.2%) patients were diagnosed with familial PHPT, comprised of five patients with MEN1, six with FHH, and one with FIHP. Other 95 patients were diagnosed with sporadic PHPT, and 13 of them had pathologically confirmed parathyroid cancer. Mean serum calcium and intact PTH levels were 11.3 ± 1.7 mg/dL and 264.6 ± 385.3 pg/mL, respectively. Seventy-seven subjects underwent gene sequencing with a targeted NGS panel, and 30 subjects underwent clinical exome sequencing. The baseline characteristics did not differ between the groups according to the NGS panel (**Supplementary Table 3**). Targeted sequencing panel detected 41 variants (one pathogenic variant, seven likely pathogenic variants, and 33 VUSs) and the other panel for clinical exome sequencing identified 11 variants (two pathogenic variants, three likely pathogenic variants and six VUSs).

**Table 1:** Baseline characteristics of study subjects.

	Study Subjects (n = 107)
Age, years	47.6 ± 16.1
Age < 40 years, n (%)	31 (29.0)
Women, n (%)	79 (73.8)
**PHPT form**	
Familial PHPT, n (%)	12 (11.2)
MEN1, n (%)	5 (4.7)
FHH, n (%)	6 (5.6)
FIHP, n (%)	1 (0.9)
Sporadic PHPT, n (%)	95 (88.8)
Benign, n (%)	82 (76.6)
Malignant, n (%)	13 (12.1)
**Gene sequencing**	
Targeted sequencing, n (%)	77 (72.0)
Clinical exome sequencing, n (%)	30 (28.0)
**Biochemistry**	
Calcium (mg/dL)	11.3 ± 1.7
Phosphorus (mg/dL)	2.8 ± 0.6
Intact PTH (pg/mL)	264.6 ± 385.3
Albumin (g/dL)	4.5 ± 0.4
Corrected Calcium for albumin (mg/dL)	11.0 ± 1.6
Ionized Calcium (mg/dL)	5.71 ± 0.72
ALP (IU/L)	125.9 ± 149.5
BUN (mg/dL)	14.4 ± 7.6
Creatinine (mg/dL)	0.8 ± 0.3
25-hydroxy vitamin D (ng/mL)	18.8 ± 9.6
24 h-urine calcium (mg/24 h)	259.2 ± 141.3
**Clinical manifestation of PHPT**	
Family history of PHPT, n (%)	4 (3.7)
Multiglandular PHPT, n (%)	4 (3.7)
Recurrent PHPT, n (%)	11 (10.3)
Persistent PHPT, n (%)	5 (4.7)

Values are shown as means with standard deviations or as numbers (%). PHPT, primary hyperparathyroidism; MEN1, multiple endocrine neoplasia type 1; FHH, familial hypocalciuric hypercalcemia; FIHP, familial isolated hyperparathyroidism; PTH, parathyroid hormone; ALP, alkaline phosphatase; BUN, blood urea nitrogen.

The variants identified by NGS are listed on **Table 2** according to their clinical diagnosis. Patients with familial PHPT or parathyroid cancer had germline mutation in *MEN1*, *CASR*, and *CDC73*, classified as pathogenic or likely pathogenic variants. The specific criteria used for each pathogenic and likely pathogenic variants are shown in **Supplementary Table 4**.

**Table 2:** Genetic variants identified by next-generation sequencing.

Variant classification	Gene	NM number	Nucleotide change	Amino acid change	Zygosity	Variant type
**I. Familial PHPT**						
**1. MEN1**						
pathogenic	*MEN1*	NM_000244.3	c.1339C>T	p.Gln447Ter	Hetero	nonsense
Likely pathogenic	*MEN1*	NM_000244.3	c.559dupG	p.Ala187GlyfsTer14	Hetero	frameshift
	*MEN1*	NM_000244.3	c.773C>T	p.Ser258Leu	Hetero	missense
	*MEN1*	NM_000244.3	c.839+1G>A		Hetero	splice site mutation
	*MEN1*	NM_000244.3	c.505del	p.Ala169ProfsTer21	Hetero	frameshift
**2. FHH**						
Likely pathogenic	*CASR*	NM_000388.3	c.658C>T	p.Arg220Trp	Hetero	missense
VUS	*AP2S1*	NM_004069.4	c.44G>A	p.Arg15His	Hetero	missense
	*APC*	NM_000038.5	c.7969G>A	p.Val2657Ile	Hetero	missense
	*CASR*	NM_000388.3	c.168G>C	p.Glu56Asp	Hetero	missense
	*CASR*	NM_000388.3	c.1287C>A	p.His429Gln	Hetero	missense
**II. Benign sporadic PHPT**						
Likely pathogenic	*CDC73*	NM_024529.4	c.685_688del	p.Arg229TyrfsTer27	Hetero	frameshift
	*LRP5*	NM_002335.3	c.731C>T	p.Thr244Met	Hetero	missense
	*SDHA*	NM_004168.2	c.151-2A>G		Hetero	splice site mutation
	*VHL*	NM_000551.3	c.24G>A	p.Trp8Ter	Hetero	nonsense
VUS	*AIP*	NM_003977.3	c.571C>T	p.Arg191Cys	Hetero	missense
	*APC*	NM_000038.5	c.8017A>G	p.Arg2673Gly	Hetero	missense
	*APC*	NM_000038.5	c.6754C>G	p.Pro2252Ala	Hetero	missense
	*CASR*	NM_000388.3	c.613C>T^a^	p.Arg205Cys	Hetero	missense
	CASR^*^	NM_000388.3	c.613C>T^a^	p.Arg205Cys	Hetero	missense
	*CDKN1A*	NM_078467.2	c.428G>A	p.Arg143Gln	Hetero	missense
	*COL1A1*	NM_000088.3	c.4262C>A	p.Ala1421Asp	Hetero	missense
	*COL1A1*	NM_000088.3	c.2090G>A	p.Arg697Gln	Hetero	missense
	*COL1A1*	NM_000088.3	c.2280T>A^b^	p.Asp760Glu	Hetero	missense
	*COL1A1*	NM_000088.3	c.2280T>A^b^	p.Asp760Glu	Hetero	missense
	*CREBBP*	NM_004380.2	c.2455G>A	p.Val819Met	Hetero	missense
	*CYP27B1*	NM_000785.3	c.589+1G>A		Hetero	splice site mutation
	*ESR1*	NM_000125.3	c.437C>A	p.Pro146Gln	Hetero	missense
	ESR2^*^	NM_001437.2	c.1541C>T	p.Pro514Leu	Hetero	missense
	*FGFR1*	NM_023110.2	c.1351_1353del^c^	p.Ser452del	Hetero	in-frame deletion
	*FGFR1*	NM_023110.2	c.1351_1353del^c^	p.Ser452del	Hetero	in-frame deletion
	*FGFR2*	NM_000141.4	c.1436A>T	p.Asp479Val	Hetero	missense
	*GATA3*	NM_001002295.1	c.1186G>A^d^	p.Ala396Thr	Hetero	missense
	*GATA3*	NM_001002295.1	c.1186G>A^d^	p.Ala396Thr	Hetero	missense
	*GATA3*	NM_001002295.1	c.706C>G	p.Pro236Ala	Hetero	missense
	*GCM2*	NM_004752.3	c.1162A>G	p.Lys388Glu	Hetero	missense
	*GHRL*	NM_001134944.1	c.71A>G	p.Gln24Arg	Hetero	missense
	*GNA11*	NM_002067.2	c.220G>A	p.Gly74Ser	Hetero	missense
	*PTH1R*	NM_000316.2	c.52G>A	p.Val18Met	Hetero	missense
	*RET*	NM_020630.4	c.1799G>A	p.Arg600Gln	Hetero	missense
	*RET*	NM_020630.4	c.833C>A	p.Thr278Asn	Hetero	missense
	*SLC34A1*	NM_003052.4	c.1238C>T	p.Thr413Ile	Hetero	missense
	*SOST*	NM_025237.2	c.159_161del	p.Asn53del	Hetero	in-frame deletion
	*TBCE*	NM_001079515.1	c.922T>A	p.Ser308Thr	Hetero	missense
	*TNFSF11*	NM_033012.3	c.205G>A	p.Ala69Thr	Hetero	missense
	*WT1*	NM_024426.4	c.296C>T	p.Ala99Val	Hetero	missense
**III. Parathyroid cancer**						
Pathogenic	*CDC73*	NM_024529.4	c.376C>T	p.Arg126Ter	Hetero	nonsense
	*CDC73*	NM_024529.4	Whole genedeletion		Hetero	Large deletion
Likely pathogenic	*CDC73*	NM_024529.4	c.687_688del	p.Arg229SerfsTer37	Hetero	frameshift
VUS	*APC*	NM_000038.5	c.5378C>G	p.Ala1793Gly	Hetero	missense
	*APC*	NM_000038.5	c.890C>T	p.Thr297Ile	Hetero	missense
	*PRKAR1A*	NM_002734.4	c.567A>C	p.Glu189Asp	Hetero	missense
	WT1^*^	NM_024426.4	c.1139G>A	p.Arg380Gln	Hetero	missense

^abcd^The same mutations found in different patients are listed separately.^*^These cases concurrently carried pathogenic or likely pathogenic variants.

PHPT, primary hyperparathyroidism; MEN1, multiple endocrine neoplasia 1; FHH, familial hypocalciuric hypercalcemia; VUS, variants of uncertain significance.

A total of 1,315 VUSs were detected in 484 genes, and we prioritized non-synonymous variants (missense variants, frameshift variants, and variants at canonical ± 1 or 2 splice sites), which were included in our candidate parathyroid gene panel (**Supplementary Table 2**). Thirty-nine VUSs were selected and are listed in **Table 2**. Among the 39 variants, three VUSs (*WT1*, *ESR2*, and *CASR*) were accompanied by pathogenic or likely pathogenic variants (*CDC73*, *LRP5*, and *VHL*, respectively). The clinical characteristics of the three cases are shown in **Table 3**.

**Table 3:** Patients with VUSs in *GCM2*, *APC*, *WT1*, *ESR2*, and *CASR* genes.

Gene	NM number	Nucleotide change	Amino acid change	Age/Sex	Diagnosis	Ca (mg/dL)	P (mg/dL)	Albumin (g/dL)	iPTH (pg/mL)	Accompanying pathogenic or likely pathogenic variants
*GCM2*	NM_004752.3	c.1162A>G	p.Lys388Glu	27/F	PHPT	10.4	2.5	4.9	84.6	No
*APC*	NM_000038.5	c.890C>T	p.Thr297Ile	25/F	Parathyroid cancer	14.7	2.2	4.8	2104.0	No
*APC*	NM_000038.5	c.5378C>G	p.Ala1793Gly	57/F	Parathyroid cancer	12.4	2.7	4.4	166	No
*APC*	NM_000038.5	c.6754C>G	p.Pro2252Ala	45/F	PHPT	10.6	2.8	4.6	90.7	No
*APC*	NM_000038.5	c.8017A>G	p.Arg2673Gly	33/F	PHPT	10.3	2.9	4.6	124.2	No
*APC*	NM_000038.5	c.7969G>A	p.Val2657Ile	37/M	FHH	10.9	2.5	4.5	73.3	No
*WT1*	NM_024426.4	c.1139G>A	p.Arg380Gln	40/F	Parathyroid cancer	12.8	2.0	4.4	192.2	Pathogenic *CDC73*
*ESR2*	NM_001437.2	c.1541C>T	p.Pro514Leu	67/F	PHPT	10.9	2.9	4.8	69.3	Likely pathogenic *LRP5*
*CASR*	NM_000388.3	c.613C>T	p.Arg205Cys	80/F	PHPT	11.3	1.8	4.4	199.4	Likely pathogenic *VHL*

iPTH, intact parathyroid hormone; PHPT, primary hyperparathyroidism; FHH, familial hypocalciuric hypercalcemia.

As *APC* and *GCM2* variants were recently reported in PHPT and parathyroid cancer, (15, 17) we analyzed the data of subjects with variants in *APC* and *GCM2* genes (**Table 3**). One of the patients had a *GCM2* variant, and was diagnosed with PHPT at the age of 27. Five subjects had *APC* variants, and their mean age at the time of diagnosis was 37.3 ± 12.0. Two out of five subjects with *APC* variants were diagnosed with parathyroid cancer.

We further analyzed the clinical characteristics of the study subjects according to their genetic tests (**Table 4**). The median age of subjects with positive test results was not significantly different from that of subjects without positive test results. Among the patients with positive test results, 38.5% had the recurrent PHPT, which was significantly higher than those without positive test results (38.5% vs. 11.7%, *p* < 0.025) We had 11 patients with recurrent PHPT in this study, and 5 of them had positive genetic test results. Four out of these five patients had either MEN1 (n=2) or parathyroid cancer (n=2). In contrast, none of the five patients with persistent PHPT had positive test result. Of the subjects with positive test results, 76.9% met at least one of the following clinical features: 1) age < 40 years, 2) family history of PHPT, 3) multiglandular PHPT, or 4) recurrent PHPT. This proportion was significantly higher in those with positive results than in those without (76.9% vs. 38.3%, *p* = 0.010). Laboratory findings, such as serum calcium and intact PTH levels, were statistically similar between the groups.

**Table 4:** Characteristics of subjects according to genetic test results.

	Subjects with positivegenetic test results (n =13)	Subjects without positive genetic test results (n = 94)	p-value
Age (years)	45.0 (26)	51.0 (24)	0.471
Calcium (mg/dL)	11.3 (2.3)	11.0 (1.3)	0.637
iPTH (pg/mL)	152.3 (194.1)	126.6 (145.3)	0.699
Meets any one of the followings	10 (76.9)	36 (38.3)	0.010
Age < 40 years, n (%)	4 (30.8)	27 (28.7)	0.555
Family history of PHPT, n (%)	2 (15.4)	2 (2.1)	0.071
Multiglandular PHPT, n (%)	1 (7.7)	3 (3.2)	0.409
Recurrent PHPT, n (%)	5 (38.5)	6 (6.4)	0.004

Values are presented as medians with interquartile ranges or numbers (%). iPTH, intact para-thyroid hormone; PHPT, primary hyperparathyroidism.

## Discussion

PHPT is a common endocrinological disorder with relatively well-established diagnosis and management. (2, 10, 19) However, knowledge about the genetic background of PHPT is limited, and genetic testing of PHPT is often overlooked in clinical practice. The development of NGS has helped advance research into the genetics of various types of endocrinological disorders. In addition, NGS is being widely used in clinical settings to detect genetic abnormalities and provide genetic counseling. We analyzed NGS data of 107 patients with PHPT and identified 3 pathogenic and 10 likely pathogenic variants. We further assessed 39 VUSs that could be related to parathyroid disease.

Genetic variants associated with parathyroid cancer and familial PHPT have been reported, and *MEN1*, *CDC73*, and *RET* mutations are known to be associated with the pathogenesis of parathyroid cancer or familial PHPT. (20) Also, in this study, patients with familial PHPT or parathyroid cancer had variants in *MEN1* and *CDC73* genes reported as pathogenic or likely pathogenic variants. We included 13 patients with parathyroid cancer, and three of them had *CDC73* mutations. Additionally, four patients with parathyroid cancer had VUSs: two *APC* variants (c.5378C>G [p.Ala1793Gly] and c.890C>T [p.Thr297Ile]), one *WT1* variant (c.1139G>A [p.Arg380Gln]), and one *PRKAR1A* variant (c.567A>C [p.Glu189As]). However, since the number of the patients with parathyroid cancer was small, it was hard to conclude the causative role of these variants.

Several somatic mutations have been identified in benign sporadic PHPTs. Somatic *MEN1* gene mutations occur in 12% to 35% of sporadic PHPT (21–24), and somatic mutations in the *CCND1* gene are also observed in 20%–40% of sporadic PHPT. (25–27) However, as the studies on germline mutations in sporadic PHPT are limited, variants found in subjects with sporadic PHPT are usually classified as VUSs. In this study, 32 non-synonymous variants in genes that could be related to parathyroid disease were classified as VUSs in patients with benign sporadic PHPT.

One of the patients had a *GCM2* variant (c.1162A>G [p.Lys388Glu]), classified as VUS. GCM2 is mainly expressed in the parathyroid gland and regulates its development. (15) Germline mutations in *GCM2* have recently been described as causative genetic alterations in FIHP. The specific genetic cause of FIHP, one of the hereditary forms of PHPT, was unclear until 2016, when Guan et al. demonstrated that *GCM2* mutation can cause FIHP. (15) They found rare variants located in the *GCM2* C-terminal conserved inhibitory domain (CCID) in 7 of the 40 kindreds with FIHP. *GCM2* variants can be found in various functional domains of the human GCM2 protein, but those with transcription-activating functions are usually located within the CCID region. (15, 18) *GCM2* variants have been reported not only in FIHP but also in sporadic PHPT. (28, 29) The prevalence of *GCM2* variants in sporadic PHPT ranges from approximately 1.5% to 26.9% depending on ethnicity, and is particularly high in the Ashkenazi Jewish population. (18, 28, 30) There are limited studies on the Asian population, and one study found that the prevalence of *GCM2* mutation with trans-activating function in Chinese PHPT patients was 1.3%. (18) In our study, one patient out of 107 had the *GCM2* variant (c.1162A>G [p.Lys388Glu]), which is located in the CCID region. This same variant was previously reported in a study of Chinese patients with sporadic PHPT. (18) They screened 232 patients diagnosed with PHPT and found two cases with the variant c.1162A>G (p.Lys388Glu) of *GCM2*. Cases with variant c.1162A>G (p.Lys388Glu) had carcinoma pathology. Considering its prevalence, location, and transcription activity, we speculated that this variant in our patient could be associated with the development of PHPT.

There were concerns that PHPT patients with *GCM2* variant could have an aggressive clinical phenotype, a high rate of multiglandular disease, and a low rate of biochemical cure. (31) Our patient with *GCM2* variant was diagnosed with PHPT during health check-up and did not have any clinical manifestations, including renal stones and low bone mass. The patient underwent right inferior parathyroidectomy, and the pathology revealed parathyroid adenoma. After the surgery, the patient achieved biochemical cure and was under routine follow-up without recurrence. However, the patient’s age at diagnosis was 27 years, indicating possible involvement of genetic components in disease development. On NGS testing, no pathogenic or likely pathogenic variant was found, and variant c.1162A>G (p.Lys388Glu) in *GCM2* was reported as VUS.

*APC* gene mutations are a well-known pathogenic mutations of familial adenomatous polyposis (FAP). Notably, germline mutations in the *APC* gene were identified in a patient with sporadic MEN1, metastatic papillary thyroid cancer, and FAP. (32) It has been suggested that *APC* gene variant might be involved in the pathogenesis of tumors in the parathyroid and thyroid glands. *APC* gene variants have also been found in parathyroid cancers. (17) The aberrant WNT/β-catenin signaling in parathyroid cancer could be due to a loss of expression or alteration of the *APC* gene (33).

In this study, two missense variants c.6754C>G (p.Pro2252Ala) and c.8017A>G (p.Arg2673Gly) of the *APC* gene were identified and classified as VUSs in patients with benign sporadic PHPT. The patients were diagnosed with PHPT at relatively young ages of 33 and 45 years. Of interest, *APC* mutations were also observed in patients with parathyroid cancer and in those with FHH. Among the 13 parathyroid cancer patients in this study, two patients carried *APC* variants (c.890C>T [p.Thr297Ile] and c.5378C>G (p.Ala1793Gly]) and did not harbor any other pathogenic or likely pathogenic variants. The other patient with *APC* variant (c.7969G>A[p.Val2657Ile]) was diagnosed with FHH and concurrently had the *AP2S1* variant, classified as VUS. These five patients with *APC* variants were relatively young at the time of diagnosis, and two were diagnosed with parathyroid cancer. It is possible that the *APC* variants in these patients are involved in the development of PHPT.

Three patients with VUSs concurrently carried pathogenic or likely pathogenic variants. One patient with a *WT1* variant (c.1139G>A [p.Arg380Gln]), reported as VUS, had a pathogenic *CDC73* mutation (c.376C>T [p.Arg126Ter]) and was diagnosed with parathyroid cancer. Pathogenic *CDC73* mutation is suspected to cause parathyroid cancer, and the *WT1* variant could be an incidental finding from NGS.

The second patient carried the likely pathogenic *LRP5* mutation (c.731C>T [p.Thr244Met]) and *ESR2* variant (c.1541C>T [p.Pro514Leu]), classified as VUS. *LRP5* mutation was not only reported in osteoporosis (34), but has also been associated with parathyroid tumors. (21, 35) In this patient, the *LRP5* mutation could have played a role in the development of PHPT and osteoporosis. In addition, based on a study reporting estrogen receptor involvement in parathyroid adenoma, (36) there is a possibility that *ESR2* variant also played a role in the development of PHPT in this case.

Lastly, a patient with *CASR* variant (c.613C>T [p.Arg205Cys]), reported as VUS, also had likely pathogenic *VHL* mutation (c.24G>A [p.Trp8Ter]). Although *VHL* mutation was reported as a likely pathogenic variant, the phenotype of *VHL* mutation is unclear in this patient, which might be due to variable expression of *VHL* mutation. (37) In contrast, *CASR* variant is associated with PHPT in previous studies. (38–40) Therefore, we speculated that although it was reported as VUS, *CASR* variant was involved in the development of PHPT and *VHL* mutation did not present its phenotype in this patient.

Genetic testing is usually indicated in some patients with PHPT who are at high risk of carrying a mutation: those with familial PHPT or parathyroid cancer (41) However, other clinical indications for genetic testing are still unclear, and the role of genetic testing in PHPT is often overlooked. In this study, 13 out of 107 study subjects (12.1%) had pathogenic or likely pathogenic variants, and 76.9% of the subjects with positive genetic test results had at least one of these clinical characteristics: 1) age < 40 years, 2) family history of PHPT, 3) multiglandular PHPT, or 4) recurrent PHPT. These clinical characteristic are generally consistent with previous studies, (3) and this implies that patients with these clinical characteristics need to undergo genetic testing.

Interestingly, 38.5% of patients with positive genetic testing had recurrent PHPT, but none of them had persistent PHPT. This different prevalence of recurrent or persistent PHPT in patients with positive test genetic testing might be due to the different clinical or genetic characteristics between the two. In our study, the patients who showed persistent PHPT were mainly due to the residual tissues of parathyroid adenoma or hyperplasia after the surgery or ectopic tissue which was not identified at the first operation. In contrast, the patients with recurrent PHPT were more likely to have genetic alterations, such as MEN1 or parathyroid cancer, so that they had the recurred disease even after the complete resection of the initial parathyroid tumor. In clinical practice, recurrent or persistent PHPT has been considered from the same point of view. However, based on the results in this study, patients with recurrent PHPT are at higher risk of having genetic alterations, thereby, should be urged to undergo genetic study. However, since this study included only small number of the patients, further study with large number of patients with recurrent or persistent PHPT is required.

The age of the subjects at the time of diagnosis did not differ between the groups according to the genetic test results. This might be due to a selection bias. Patients with classical sporadic form of PHPT and aged above 50 years would be less likely to undergo genetic testing and thus were not included in this study. In contrast, young patients were easily suspected to have genetic abnormalities, underwent genetic testing, and were enrolled in this study retrospectively. Therefore, there might be selection bias and the difficulty in determining the age difference between the groups with and without positive test results. In addition, the number of subjects with positive genetic test results was relatively small, leading to statistically insignificant results.

There are several other limitations to this study. This study included heterogeneous groups of PHPT, familial PHPT, benign sporadic PHPT, and parathyroid cancer. However, the number of subjects with familial PHPT and parathyroid cancer may not be sufficient to detect novel gene mutations. Second, the NGS panel used in this study did not include all candidate genes related to parathyroid disease. Lastly, because two different sequencing panels were used among the study patients, it might have affected the results.

In this study, we analyzed the NGS results of patients with PHPT. We speculated that although some variants were reported as VUSs, they could be associated with the development of the disease. In particular, as well as patients with familial PHPT or parathyroid cancer, which are classical indications for NGS, patients with young age or recurrent disease should be urged to undergo genetic testing. Advances in genetics and the declining cost of genetic testing may lead to its wider utilization in the future, thus helping in the diagnosis and management of PHPT patients and their relatives. We believe that this study provides insights into the genetic background of PHPT and provides a better approach for genetic counseling. Further studies are warranted to investigate the genetic abnormalities in PHPT pathogenesis and the role of NGS in PHPT in clinical practice.

## Data Availability Statement

The original contributions presented in the study are included in the article/**Supplementary Material**. Further inquiries can be directed to the corresponding author.

## Ethics Statement

The studies involving human participants were reviewed and approved by Institutional Review Board of Severance Hospital, Yonsei University Health System, Seoul, Korea. Written informed consent for participation was not required for this study in accordance with the national legislation and the institutional requirements.

## Author Contributions

Conceptualization: H-SP, NH, and YR. Methodology: H-SP, NH, DW, and YR. Formal analysis: H-SP and YR. Investigation: H-SP, YL, NH, and YR. Writing—original draft preparation: H-SP, and YL. Writing—review and editing: H-SP, YL, NH, DW, and YR. Supervision: YR. All authors have read and agreed to the published version of the manuscript.

## Funding

This study was supported by Hanim Precision Medicine Center of Yonsei University Health System under Grant number (6-2021-0208).

## Conflict of Interest

The authors declare that the research was conducted in the absence of any commercial or financial relationships that could be construed as a potential conflict of interest.

## Publisher’s Note

All claims expressed in this article are solely those of the authors and do not necessarily represent those of their affiliated organizations, or those of the publisher, the editors and the reviewers. Any product that may be evaluated in this article, or claim that may be made by its manufacturer, is not guaranteed or endorsed by the publisher.

## Supplementary Material

Supplementary Table 1 Gene panel list: targeted gene sequencing. Genes which are included in our candidate gene list (**Supplementary Table 2**) are shown in yellow box.

Supplementary Table 2 List of candidate genes related to parathyroid diseases.

Supplementary Table 4 The criteria used for each pathogenic and likely pathogenic variants.
