# Supplementary material for: Germline Mutations Related to Primary Hyperparathyroidism Identified by Next-Generation Sequencing
Source: Front Endocrinol (Lausanne). 2022 Apr 28;13:853171. doi: 10.3389/fendo.2022.853171 (PMC9109676; doi:10.3389/fendo.2022.853171)
Supplement: Supplementary Table 1 — Gene panel list: targeted gene sequencing. Genes which are included in our candidate gene list ( Supplementary Table 2 ) are shown in yellow box. [file DataSheet_1.zip › Supplementary Table 3.docx]

|  | **Targeted sequencing**  **(n = 77)** | **Clinical exome sequencing**  **(n = 30)** | ***p*-value** |
| --- | --- | --- | --- |
| Age, years | 51.0 (29) | 45.5 (16) | 0.471 |
| Age < 40 years, n (%) | 24 (31.2) | 7 (23.3) | 0.290 |
| Women, n (%) | 55 (71.4) | 24 (80.0) | 0.466 |
| **PHPT form** |  |  |  |
| Familial PHPT, n (%) | 10 (13.0) | 2 (6.7) | 0.503 |
| MEN1, n (%) | 4 (5.2) | 1 (3.3) | >0.999 |
| FHH, n (%) | 5 (6.5) | 1 (3.3) | >0.999 |
| FIHP, n (%) | 1 (1.3) | 0 (0.0) | >0.999 |
| Sporadic PHPT, n (%) | 67 (87.0) | 28 (93.3) | 0.503 |
| Benign, n (%) | 57 (74.0) | 25 (83.3) | 0.446 |
| Malignant, n (%) | 10 (13.0) | 3 (10.0) | >0.999 |
| **Biochemistry** |  |  |  |
| Calcium (mg/dL) | 11.1 (1.6) | 11.3 (1.2) | 0.637 |
| Phosphorus (mg/dL) | 2.7 (0.7) | 2.8 (1.0) | 0.070 |
| Intact PTH (pg/mL) | 134.0 (155.2) | 161.0 (244.0) | 0.699 |
| Albumin (g/dL) | 4.5 (0.4) | 4.6 (0.3) | 0.205 |
| Corrected Calcium for albumin (mg/dL) | 10.7 (1.7) | 10.9 (1.3) | 0.480 |
| Ionized Calcium (mg/dL) | 5.6 (0.6) | 5.6 (0.6) | 0.223 |
| **Clinical manifestation of PHPT** |  |  |  |
| Family history of PHPT, n (%) | 1 (1.3) | 3 (10.0) | 0.066 |
| Multiglandular PHPT, n (%) | 4 (5.2) | 0 (0.0) | 0.575 |
| Recurrent PHPT, n (%) | 8 (10.4) | 3 (10.0) | >0.999 |
| Persistent PHPT, n (%) | 5 (6.5) | 0 (0.0) | 0.319 |

Supplementary Table 3: The baseline characteristics of study subjects according to the gene sequencing panel

Values are presented as medians with interquartile ranges or numbers (%). PHPT, primary hyperparathyroidism; MEN1, multiple endocrine neoplasia type 1; FHH, familial hypocalciuric hypercalcemia; FIHP, familial isolated hyperparathyroidism; PTH, parathyroid hormone.
